# Alcohol consumption before pregnancy causes detrimental fetal development and maternal metabolic disorders

**DOI:** 10.1038/s41598-020-66971-1

**Published:** 2020-06-22

**Authors:** Yoo Jeong Lee, Ji Yeon Kim, Dae Yeon Lee, Keon Jae Park, Gyu Hee Kim, Jeong Eun Kim, Gu Seob Roh, Joong Yeon Lim, Seul Koo, Nam Kyoo Lim, Hyun Young Park, Won-Ho Kim

**Affiliations:** 1 Division of Cardiovascular Disease, Center for Biomedical Sciences, Korea National Institute of Health, Cheongju, Chungbuk 28159 Republic of Korea; 2 Division of Endocrine and Metabolic Disease, Center for Biomedical Sciences, Korea National Institute of Health, Cheongju, Chungbuk 28159 Republic of Korea; 3 Department of Biotechnology, Korea University, Seoul, Republic of Korea; 4 Department of Anatomy, College of Medicine, Institute of Health Sciences, Gyeongsang National University, Jinju, Gyeongnam Republic of Korea

**Keywords:** Translation, Biomarkers, Risk factors

## Abstract

Alcohol consumption before or during pregnancy poses serious health risks to the fetus; however, the underlying mechanisms involved remain obscure. Here, we investigated whether ethanol consumption before pregnancy affects maternal or fetal health and whether pharmacological inhibition of CYP2E1, a major ethanol oxidation enzyme, by 4-methylpyrazole (4-MP) has therapeutic effects. We found that ethanol consumption (5%) 2 weeks before pregnancy resulted in a decrease in the number of viable fetuses and abnormal fetal development, and these effects were accompanied by impaired maternal glucose homeostasis and hepatic steatosis during pregnancy. Neonates of ethanol-fed mice had postnatal macrosomia and significantly decreased growth rates during the lactation period. However, treatment with 4-MP, a CYP2E1 inhibitor, markedly ameliorated the reduction in insulin action and glucose disposal responsiveness in the livers of ethanol-fed mice. Blockage of CYP2E1 significantly reduced the alteration in hepatic lipid deposition, fatty acid oxidation, mitochondrial energy status, and macrophage infiltration observed in ethanol-fed mice. Finally, there was a positive correlation between postnatal macrosomia or growth retardation and increased inflammatory responses. Collectively, our study suggests that even moderate ethanol intake may be detrimental to fetal development and may cause growth retardation through maternal metabolic disorders.

## Introduction

Maternal alcohol consumption during pregnancy has been associated with the disruption of metabolic pathways in the mother, thus impairing fetal development and postnatal growth^1,2^. Several studies comparing prepregnancy and pregnancy drinking have shown that approximately half of all women stop drinking once pregnancy is recognized, and among the women who do not completely stop drinking during pregnancy, the majority actually reduce their drinking to much lower weekly levels^3,4^. Although the rates of alcohol use among women vary due to the cultural differences of each country, women’s alcohol use may affect their sexual behavior and is associated with the risk of unplanned pregnancy^5,6^. Importantly, women with an unplanned pregnancy may continue to drink at prepregnancy levels during the very early weeks of pregnancy before pregnancy recognition^4^. Alcohol consumption during prepregnancy may be closely associated with unintended fetal alcohol exposure, and it may be a causal factor for detrimental effects on maternal and fetal health.

Pregnancy is a dynamic state that involves a series of small, continuous physiological changes and adjustments that affect the metabolism of all nutrients. Nutrient metabolism is continuously adjusted throughout pregnancy, and the changes are driven by hormonal changes, fetal demands, and maternal nutrient supply. During pregnancy, fasting glucose levels progressively decrease as gestation progresses, whereas hepatic glucose production increases in late pregnancy;^7^ this is consistent with the increase in fasting insulin and is linked to maternal hepatic insulin insensitivity. Insulin resistance is observed in the mother during pregnancy, is attributed to the increased concentration of several placental hormones, and may facilitate the delivery of energy substrates to the fetus^8^. The first half of pregnancy is primarily a time of preparation for the demands of rapid fetal growth that occurs late in pregnancy. Additionally, energy metabolism and fetal growth depend on the prepregnancy energy status of the mother and the quality of the living conditions during pregnancy^9^. In fact, pregnancy is referred to as an alteration in maternal metabolic homeostasis and may be vulnerable to various stressors, such as obesity, smoking, and alcohol consumption. Although alcohol consumption is associated with low food intake and low blood glucose levels in rodents^10–12^, drinking before pregnancy, in particular, may cause maternal metabolic disorders and may impair fetal development by altering the first maternal adjustment of nutrient metabolism. Additionally, drinking before pregnancy may cause indirect changes in maternal nutrient substrates, which can affect early embryo development, such as oocyte maturation, fertilization, early epigenetic reprogramming, and cell division and differentiation^13,14^. Although evidence regarding the harmful effects of drinking during pregnancy on maternal and fetal health has been continuously accumulating, little is yet known about the exact influence of maternal drinking before pregnancy on fetal development and growth. The majority of ethanol metabolism occurs in the liver, and several distinct pathways contribute to ethanol metabolism. Alcohol dehydrogenase (ADH), catalase, and cytochrome P450 2E1 (CYP2E1) are involved in its conversion to acetaldehyde, and aldehyde dehydrogenases (ALDHs) convert acetaldehyde to acetate. Among these enzymes, CYP2E1 is the major enzyme that catalyzes ethanol oxidation in the mitochondria of the liver. CYP2E1 can inadvertently produce ROS in its active site when catalysis is not correctly coordinated, resulting in potential lipid peroxidation as well as protein and DNA oxidation. Therefore, CYP2E1 expression may be important for the negative physiological effects observed in a number of disease states, and its expression levels have been correlated with a variety of dietary and physiological factors, such as ethanol consumption, diabetes, and obesity^15^. While ethanol metabolic pathways in the liver are relatively well established, considerably less is understood regarding enzymes and pathways involved in ethanol metabolism during pregnancy progression. Therefore, we hypothesized that ethanol consumption-mediated oxidative stress may further exacerbate the changes in maternal metabolic homeostasis observed during pregnancy, resulting in impaired maternal metabolism and fetal growth. Hence, the aim of this study was to explore whether drinking before pregnancy affects maternal metabolic homeostasis and fetal growth during pregnancy and to determine the exact regulatory mechanisms involved in poor pregnancy outcomes.

## Results

### Maternal drinking before pregnancy reduces the pregnancy rate and impairs fetal development

**Figure 1:**
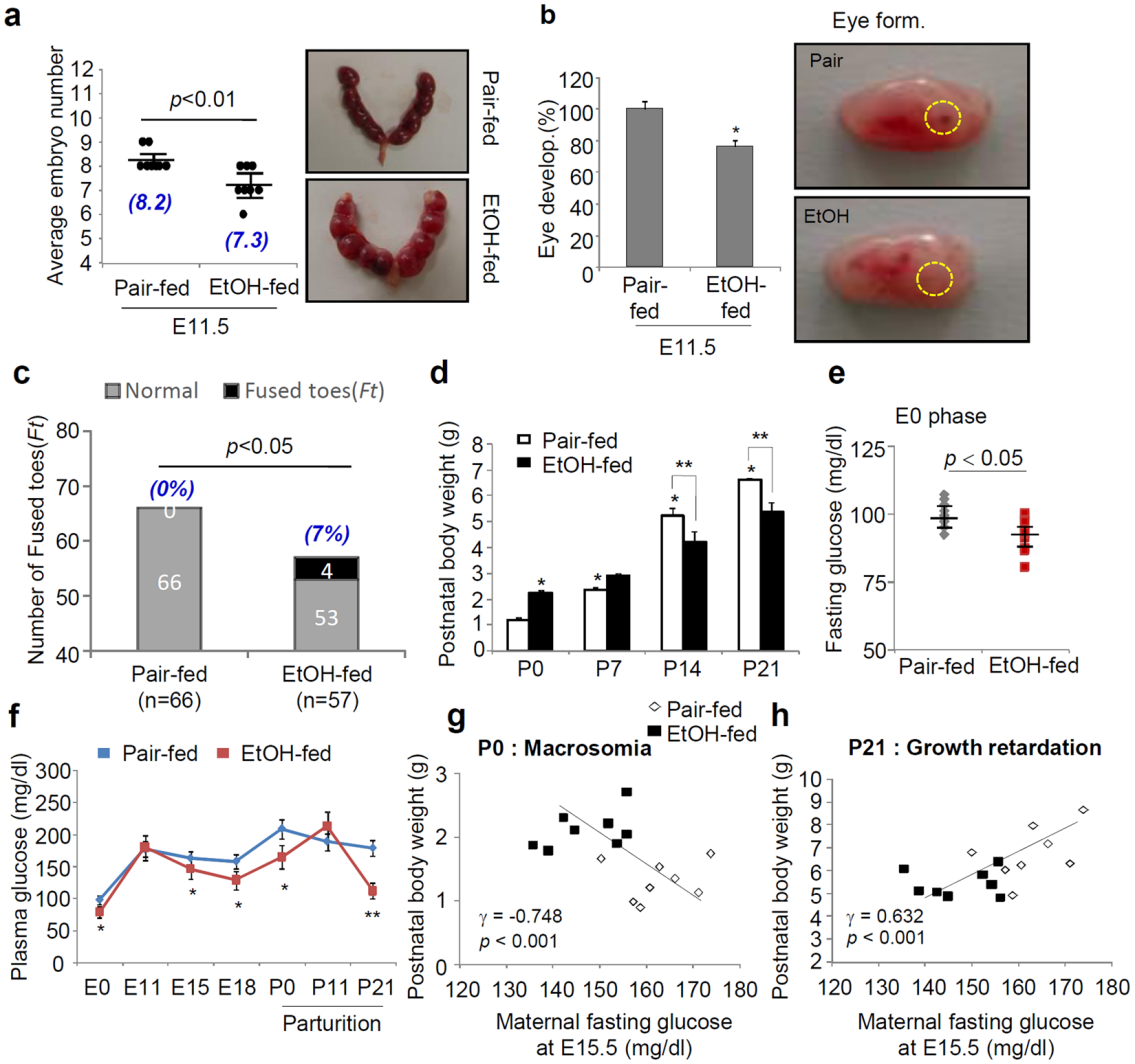
Maternal alcohol consumption prior to pregnancy impairs embryonic development and hepatic steatosis. Six-week-old female C57BL/6 J mice (n = 8 dams/group) were exposed to a liquid control or ethanol diet for 2 weeks and were impregnated thereafter. (**a**) Quantification (left) and representative imaging (right) of embryo number at E11.5 in ethanol-fed mice compared to in pair-fed mice (*p* < 0.01; n = 8 dams/group, n = 6 to 9 embryos per dam). The numbers (blue letters) at the bottom of the bar graph represent the average embryo number. (**b**) Eye development rate (left) and representative imaging of fetuses (right) in E11.5 mice (*p < 0.01; n = 8 dams/group, n = 6 to 9 embryos per dam). The yellow circle is the region of fetal eye formation. (**c**) Comparison of the number of normal healthy and fused toes (*Ft*) in offspring (postnatal (P0) phase) according to maternal alcohol exposure before pregnancy. Differences between categorical variables were analyzed (*p* < 0.05 vs. pair-fed mice; n = 8 dams/group, the number of offspring, 66 normal healthy and 0 *Ft* for pair-fed mice and 53 normal healthy and 4 *Ft* for ethanol-fed mice). The numbers (blue letters) at the top of the bar graph represent the rate of fused toes in offspring obtained in each group. (**d**) Comparison of postnatal body weight in neonates of ethanol-fed and pair-fed mice (n = 8 dams/group). Values of postnatal body weight are the mean of all offspring per dam mouse (n = 6 to 9 pups/dam); *p < 0.05 vs. offspring of pair-fed mice at P0, **p < 0.01 vs. offspring of pair-fed mice at each P14 and P21 phase, respectively from post hoc analysis for the ANOVA; *p* < 0.001 from the ANOVA. (**e**) Maternal fasting glucose levels in the E0 phase (*p* < 0.05; n = 8 dams/group). (**f**) Fasting blood glucose levels were measured in dams during pregnancy (E0 ~ E18) and the postnatal lactation period (P0 ~ P21) (n = 8 dams/group). *p < 0.05, **p < 0.01 vs. pair-fed mice, respectively from post hoc analysis for the ANOVA; *p* < 0.001 from the ANOVA. (**g**,**h**) Correlation between P0 macrosomia (left, g) or P21 growth retardation (right, h) and maternal fasting glucose levels at E15.5. Pearson coefficients (γ) and *p* values are given. Each postnatal body weight is the mean of pups per dam (n = 6 to 9 pups/dam). Values are means ± SEMs. Data in (**a**,**b** and **e**) were analyzed using two-tailed unpaired Student’s *t* test. Data in g (the number and rate) were analyzed by Fisher’s exact test and unpaired t-test. Data in **d** and **f** were analyzed by two-way repeated measures ANOVA with Tukey’s post hoc multiple comparison test. EtOH, ethanol; E11.5, embryonic day 11.5; P0, postnatal day 0; SEM, standard error of the mean.

To examine whether maternal drinking before pregnancy may affect pregnancy or birth rate and fetal development, 6-week-old female C57BL/6 J mice were exposed to a liquid ethanol diet (5%) for 2 weeks before they were impregnated (Fig. S1a). Compared with pair-fed mice, ethanol-fed mice experienced a decrease in the pregnancy rate, but there were no changes in the survival rate (Fig. S1b). Although the body weights of mice pre-exposed to ethanol before pregnancy were lower than those of pair-fed mice, there were no differences in maternal weight gain during pregnancy (Fig. S1c). The average embryo number at embryonic day 11.5 (E11.5) in ethanol-fed mice was lower than that of pair-fed mice and was accompanied by a reduction and/or delay in eye development in the embryos (Fig. 1a,b). Toe deformities defined with fused toes (*Ft*) were observed in neonates from the ethanol-fed mice group (53 normal healthy and 4 *Ft* in offspring), while no abnormalities were observed in neonates of the pair-fed mice group (Fig. 1c). Intriguingly, neonatal (P0) birth weight was approximately 2-fold higher in pups of the ethanol-fed mice than in those of the pair-fed mice, suggesting the development of macrosomia, whereas after postnatal days (P) 14 and 21, the increase in bodyweight was less in the neonates of ethanol-fed mice, suggesting an increase in growth retardation (Fig. 1d). In ethanol-fed mice, the maternal fasting blood glucose levels were decreased at E0, and low glucose levels were continuously exhibited both during pregnancy and after parturition (Fig. 1e,f). There was a significant negative correlation between neonatal P0 body weight (macrosomia) and maternal fasting glucose levels at E15.5 in ethanol-fed mice (Fig. 1g,h). Conversely, the growth retardation observed in the pups of ethanol-fed mice at P21 correlated with the lower fasting glucose levels of the mother.

### Ethanol consumption prior to pregnancy leads to hepatic steatosis during gestation

**Figure 2:**
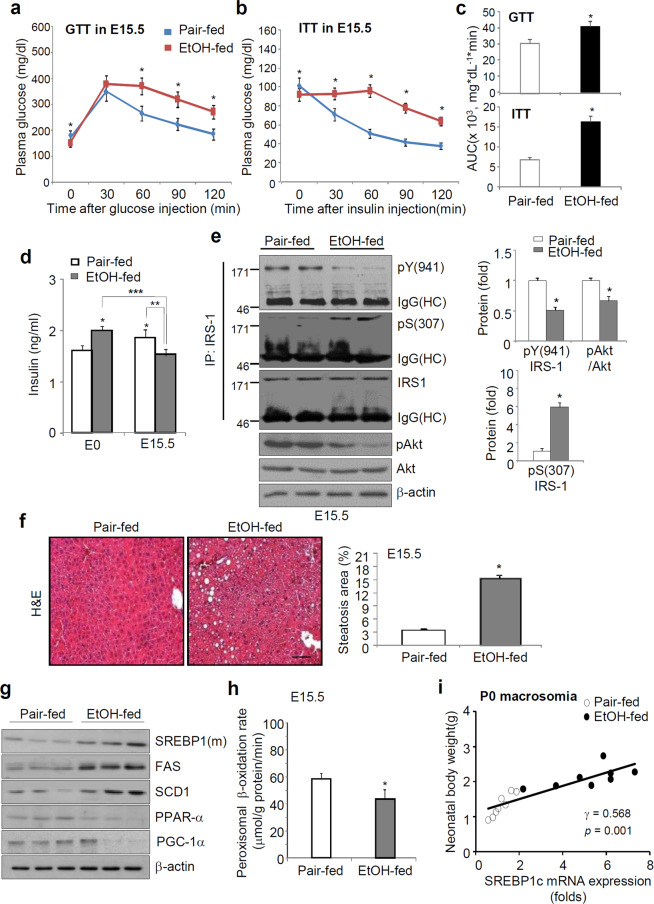
Hepatic steatosis correlates with impaired fetal development and metabolic disorders. (**a,b**) Plasma glucose levels during the glucose tolerance test (GTT, n = 8 dams/group) (**a**) and insulin tolerance test (ITT, n = 4 dams/group) (**b**) in pregnant mice carrying E15.5 embryos. *p < 0.01 vs. pair-fed mice from post hoc analysis for the ANOVA; *p* < 0.001 from the ANOVA. (**c**) Area under the curve (AUC) analysis for GTT (**a**) and ITT (**b**). *p < 0.01. (**d**) Changes in *p*lasma insulin levels at E0 and E15.5 of mice with or without ethanol consumption before pregnancy (*p < 0.05 vs. pair-fed mice at E0; **p < 0.01 vs. pair-fed mice at E15.5; ***p < 0.001 vs. EtOH-fed mice at E0; n = 8 dams/group). (**e**) Re*p*resentative immunoblots of insulin receptor substrate 1 (IRS-1) immunoprecipitates and total lysates (top) and densitometric quantification of blots (lower, n = 8). *p < 0.01 vs. pair-fed mice at E15.5. (**f**) H&E staining of liver tissue and steatosis score were measured (scale bar, 50 μm; *p < 0.01, n = 8). (**g**) Hepatic expression of proteins related to fatty acid oxidation and lipid accumulation by Western blots. Data for densitometry quantification of blots are shown in Fig. S2d(top panel). (**h**) Comparison of peroxisomal β-oxidation rate at E15.5 (*p < 0.05 vs. pair-fed mice; n = 8). (**i**) Correlation between SREBP1c mRNA expression and P0 macrosomia. Neonatal body weight is the mean body weight of the pups (n = 6 to 9 pups/mouse). Correlation coefficients (*r*) and *p* values are given. Data are expressed as the mean ± SEM. Data in **a** and **b** were analyzed by two-way repeated measures ANOVA with Tukey’s post hoc test. Data in **d** were analyzed by two-way ANOVA with Tukey’s post hoc multiple comparison test. Data in **c,e,f**, and h were analyzed using unpaired Student’s *t* test. pY, phosphorylated tyrosine; pS, phosphorylated serine; IP, immunoprecipitation.

Glucose tolerance tests (GTTs) revealed that ethanol-fed mice displayed a greater glucose homeostasis impairment than pair-fed mice, which was accompanied by a significant decrease in insulin-stimulated glucose disposal ability (Fig. 2a–c). At the E0 phase, ethanol-fed mice exhibited higher plasma fasting insulin levels than pair-fed mice, whereas E15.5 ethanol-fed mice displayed markedly lower plasma insulin levels than pair-fed mice (Fig. 2d); this finding correlated with the reduction in insulin expression in the islet cells of ethanol-fed mice (Fig. S2a). We also clarified that the plasma insulin levels during the GTTs performed at E15.5 were significantly lower in the ethanol-fed mice than in the pair-fed mice (Fig. S2b). Glucose-stimulated insulin secretion (GSIS) levels were much lower in the islets isolated from ethanol-fed mice than those isolated from pair-fed mice (Fig. S2c). Consistent with the decreased insulin sensitivity, inactive IRS-1(Ser-307) phosphorylation was increased in the maternal livers (E15.5) of ethanol-fed mice, whereas phosphorylation of active IRS-1(Tyr-941) and AKT, a downstream signaling pathway of IRS-1, was significantly decreased (Fig. 2e). Also, a prominent accumulation of lipid droplets was observed in ethanol-fed mice (Fig. 2f) and correlated with a significant increase in hepatic protein and mRNA expression of lipogenic genes, sterol regulatory element binding protein 1c (SREBP1c), fatty acid synthase (FAS), and stearoyl-CoA desaturase-1 (SCD-1), as was confirmed by immunohistochemical analysis (Fig. 2g and S2d,e). Conversely, the expression levels of peroxisome proliferator-activated receptor-α (PPARα) and PPAR-γ coactivator 1α (PGC-1α), genes related to fatty acid oxidation, were decreased in ethanol-fed mice, resulting in a decreased rate of peroxisomal fatty acid oxidation (Fig. 2h). The increase in SREBP1c mRNA expression was negatively and positively correlated with peroxisomal β-oxidation (Fig. S2f) and neonatal body weight at P0 (macrosomia) (Fig. 2i), respectively. Hepatic and plasma levels of triglyceride and cholesterol as well as glycerol, nonesterified fatty acids, IL-6, and TNF-α were potently increased during pregnancy in ethanol-fed E15.5 mice, whereas the adiponectin level, a predictive marker of type 2 diabetes (T2DM), was markedly decreased (Table S1). The increased hepatic triglyceride levels were correlated with neonatal macrosomia (P0) and postnatal growth retardation (P21) (Fig. S2g,h).

### Ethanol-mediated maternal metabolic disorders and abnormal fetal development related to ethanol metabolism

**Figure 3:**
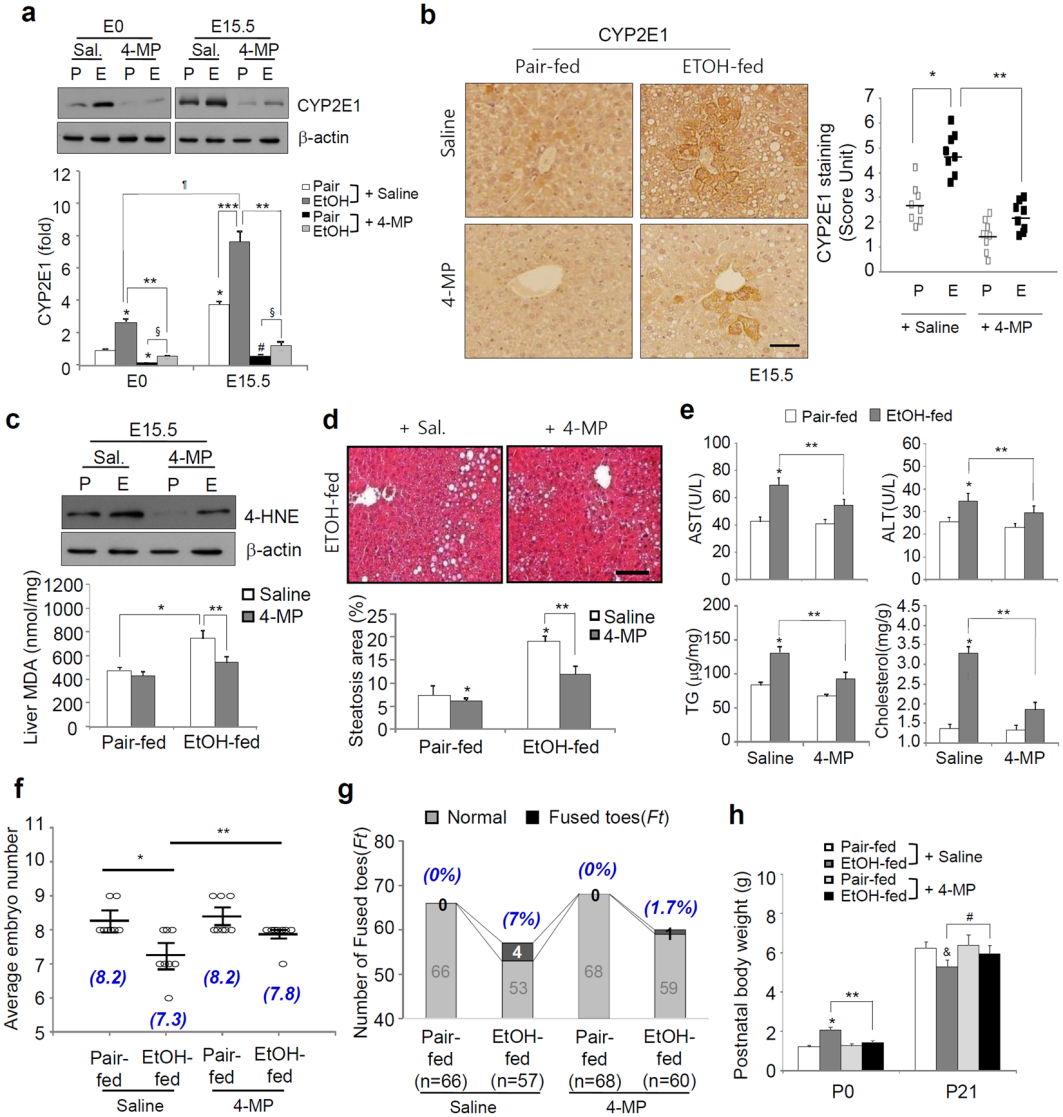
4-MP administration inhibits maternal liver injury and impaired fetal development. The mice were exposed to a liquid ethanol diet for 2 weeks, and simultaneously, 4-MP (10 mg/kg, i.p., n = 8 dams/group) was administered three times per week for 2 weeks. (**a**) Representative Western blots using an antibody against CYP2E1 in total lysates of the maternal livers of mice at E0 and E15.5 of gestation (top) and densitometry quantification relative to β-actin (bottom). Effects of 4-MP at the E0 (left) and E15.5 (right) period: *p < 0.05 vs. pair-fed mice with saline at E0, **p < 0.01 vs. ethanol-fed mice with saline at E0 or E15.5, respectively, ***p < 0.001 and ^#^*p* < 0.001 vs. pair-fed mice with saline at E15.5, ^§^*p* < 0.001 vs. pair-fed mice with 4-MP at E0 and E15.5, respectively, *p* < 0.001 vs. EtOH-fed mice with saline at E0 (n = 8 dams/group). (**b**) IHC analysis of CYP2E1 in the livers of E15.5 mice (scale bar, 50 μm) (left) and staining hepatocytes were scored (right). (**c**) Western blotting for 4-HNE (top panel) and measuring malondialdehyde (MDA) levels (bottom panel) were performed in the livers of E15.5 mice. (**d**) H&E staining (scale bar, 50 μm) of liver sections after exposure to ethanol and/or 4-MP was performed, and the steatosis area (%) was measured. (**e**) The inhibitory effects of 4-MP on serum levels of AST, ALT, hepatic triglyceride, and cholesterol at E15.5. (**f**) Restoring of average embryonic number by 4-MP (n = 6 to 9 pups/mouse). The numbers (blue letters) at the bottom of the bar graph represent the average embryo number. (**b–f**) *p < 0.05 vs. pair-fed mice with saline, **p < 0.01 vs. ethanol-fed mice with saline. (**g**) 4-MP reduces the number of offs*p*ring with fused toes (*Ft*) increased in ethanol-fed mice. Differences between categorical variables were analyzed by Fisher’s exact test (*p* < 0.05; n = 8 dams/group; the number of offspring= 68 normal healthy and 0 *Ft* for 4-MP-ex*p*osed pair-fed mice and 59 normal healthy and 1 *Ft* for ethanol-fed mice). The numbers (blue letters) at the top of the bar graph represent the rate of fused toes in offspring obtained in each group. (**h**) 4-MP ameliorates the alterations in postnatal body weight at P0 and P21. Values of postnatal body weight are the mean of pups (n = 6 to 9 pups/mouse; n = 8 dams/group). Effects of 4-MP on macrosomia at P0 (left): *p < 0.05 vs. pair-fed mice with saline, **p < 0.01 vs. ethanol-fed mice with saline; effects of 4-MP on growth retardation at P21 (right): ^&^*p* < 0.001 vs. pair-fed mice with saline, ^#^*p* < 0.001 vs. ethanol-fed mice with saline. Data are expressed as the mean ± SEM. Data in **a–f** and **h** were analyzed by two-way ANOVA with Tukey’s post hoc multiple comparison test. Data in **g** (the number and rate) were analyzed by Fisher’s exact test and unpaired t-test. P, Pair-fed; E, Ethanol-fed.

To define the effects of ethanol metabolism, ethanol-fed mice were administered 4-MP, a CYP2E1 inhibitor^16^, for 2 weeks (Fig. S1a). CYP2E1 expression increased in ethanol-fed mice at both E0 and E15.5 and was significantly reduced by 4-MP (Fig. 3a,b). 4-MP markedly reduced 4-hydroxy-2-nonenal (4-HNE) and malondialdehyde (MDA), markers of oxidative stress and lipid peroxidation^17,18^, increased in the livers of ethanol-fed mice (Fig. 3c). 4-MP injection caused a significant reduction in hepatic steatosis, and the alanine aminotransferase (ALT) or aspartate aminotransferase (AST) levels increased in ethanol-fed mice and correlated with a substantial reduction in the hepatic triglyceride (TG) and cholesterol levels (Fig. 3d,e). Importantly, CYP2E1 inhibition by 4-MP cotreatment ameliorated the reduction in the average embryo numbers and increased the number and rate of offspring with fused toes by ethanol consumption, correlating with a significant amelioration of increased neonatal macrosomia and offspring growth retardation (Fig. 3f–h).

### Blockage of CYP2E1 ameliorates glucose tolerance and insulin insensitivity in ethanol-fed mice

**Figure 4:**
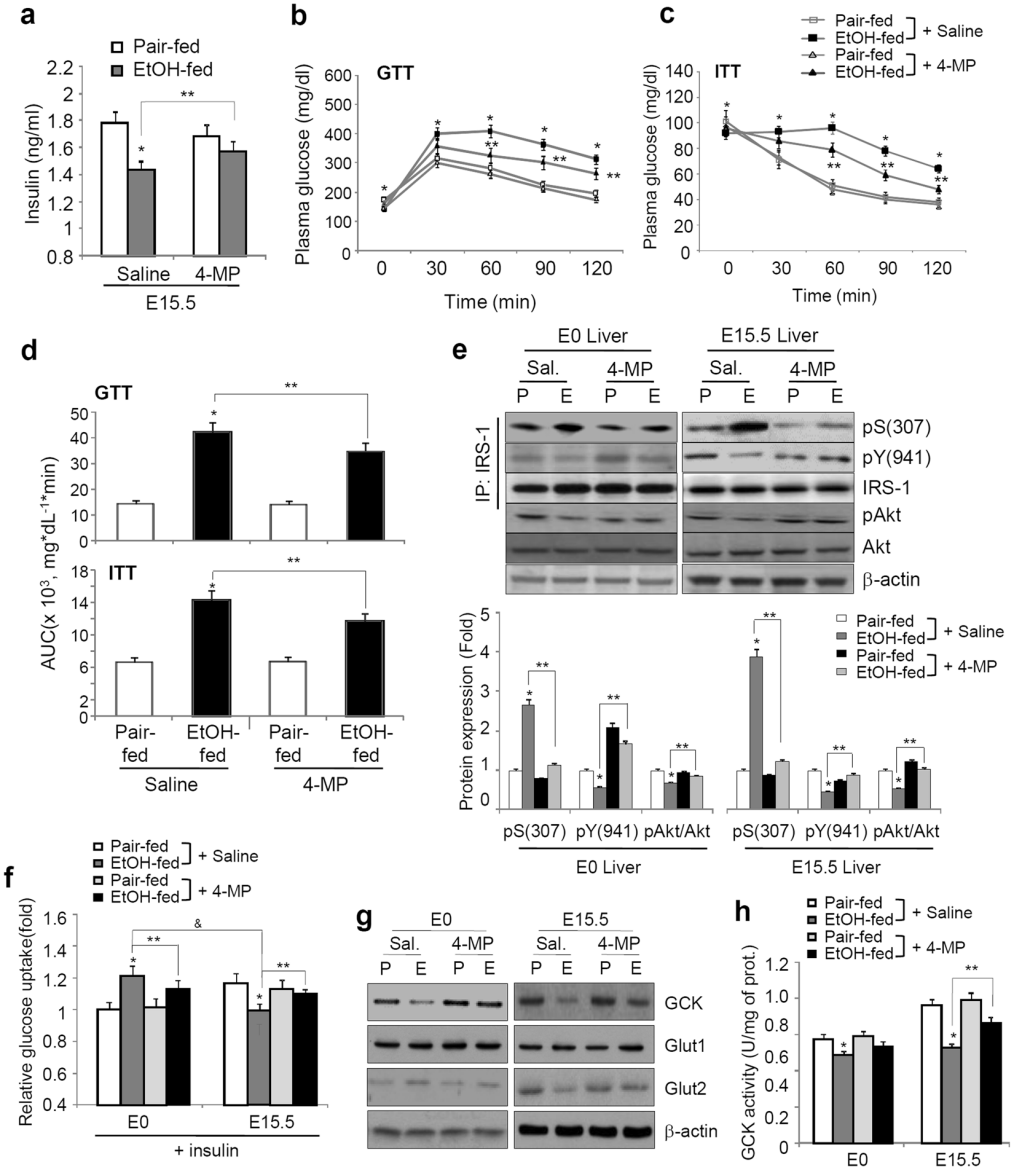
4-MP administration inhibits maternal liver injury. The mice were exposed to a liquid ethanol diet for 2 weeks, and simultaneously, 4-MP (10 mg/kg, i.p., n = 8 dams/group) was administered three times per week for 2 weeks. (**a**) Restoring of plasma insulin levels by 4-MP. *p < 0.05 vs. pair-fed mice with saline, **p < 0.01 vs. EtOH-fed mice with saline (n = 8/group). (**b**,**c**) 4-MP ameliorates glucose disposal ability (GTT, n = 8) and ethanol-mediated insulin intolerance in pregnant mice carrying E15.5 (ITT, n = 4). *p < 0.01 vs. pair-fed mice with saline, **p < 0.05 vs. EtOH-fed mice with saline from post hoc analysis for the ANOVA; *p* < 0.005 from the ANOVA. (**d**) AUC analysis for GTT (**b**) and ITT (**c**). *p < 0.01, **p < 0.05. (**e**) Representative immunoblots of insulin signaling pathway-related proteins in the liver tissues of E0 and E15.5 mice (top panel) and densitometry quantification (bottom panel). *p < 0.05, **p < 0.05. (**f**) Effects of 4-MP on relative glucose uptake in primary he*p*atocytes from E0 and E15.5 mice. *p < 0.05 vs. pair-fed mice with saline at both E0 and E15.5, **p < 0.05 vs. EtOH-fed mice with saline at both E0 and E15.5, ^&^ *p* < 0.001 vs. EtOH-fed mice with saline at E0 (n = 8 dams/group). (**g**) Western blotting for GCK, Glut1, and Glut2. (**h**) GCK activity in the livers of E0 and E15.5 mice exposed to ethanol and/or 4-MP. *p < 0.05 vs. pair-fed mice with saline at both E0 and E15.5, **p < 0.05 vs. EtOH-fed mice with saline at E15.5 (n = 8 dams/group). Data are expressed as the mean ± SEM. Data in **a,d–f** and **h** were analyzed by two-way ANOVA with Tukey’s post hoc multiple comparison test. Data in **b** and **c** were analyzed by two-way repeated measures ANOVA with Tukey’s post hoc test. P, Pair-fed; E, Ethanol-fed.

Consistently, 4-MP rescued the serum insulin levels reduced in ethanol-fed mice (Fig. 4a) and caused a greater responsiveness in glucose disposal abilities, as determined by a GTT and an insulin tolerance test (ITT) (Fig. 4b–d), followed by the rescue of the inactivated IRS-1/Akt pathway (Fig. 4e). The relative rate of insulin-stimulated glucose uptake was significantly decreased in primary hepatocytes isolated from the maternal livers of ethanol-fed mice at E15.5 (Fig. 4f), thus reflecting the significant reduction in the expression of glucokinase (GCK) and glucose transporter 2 (Glut2) and in GCK activity (Fig. 4g,h), which was alleviated by 4-MP administration. In contrast, at E0, the insulin-stimulated glucose uptake ability and Glut2 expression were increased in ethanol-fed mice, but the expression and activity of GCK, a major intracellular regulator of glucose metabolism, were significantly decreased in ethanol-fed mice at E0, suggesting the specific effects of intracellular glucose metabolism on ethanol-mediated maternal metabolic disorders.

### Ethanol metabolism-mediated ROS production is related to maternal mitochondrial dysfunction

**Figure 5:**
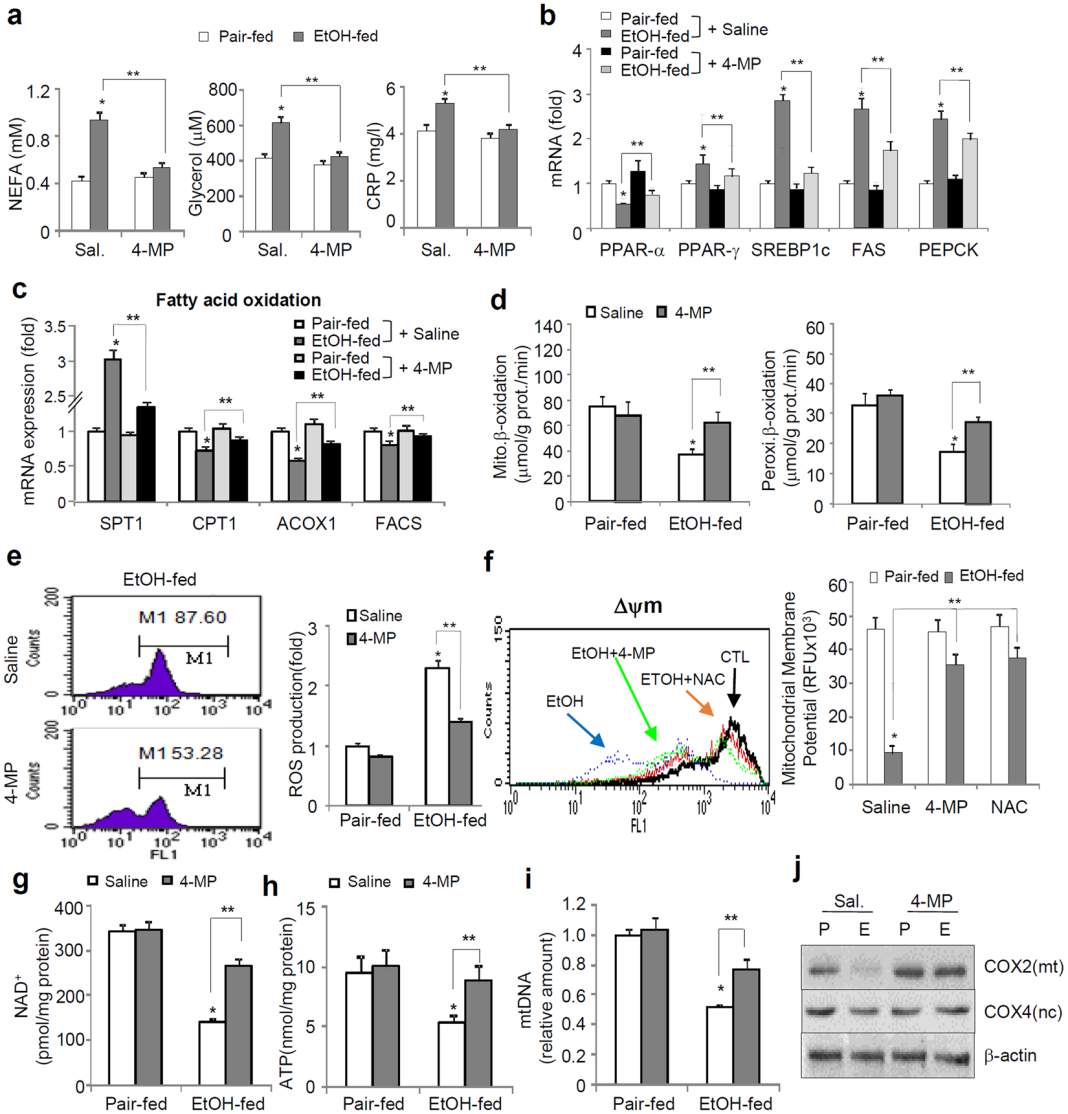
4-MP administration inhibits ethanol-induced alteration of the hepatic metabolic rate. The mice were exposed to a liquid ethanol diet for 2 weeks, and simultaneously, 4-MP (10 mg/kg, i.p., n = 8 dams/group) was administered three times per week for 2 weeks. (**a**) Plasma levels of nonesterified fatty acid (NEFA), glycerol, and C-reactive protein (CRP). *p < 0.05 vs. pair-fed mice with saline, **p < 0.01 vs. EtOH-fed mice with saline (n = 8 dams/group). (**b**,**c**) Real-time PCR for PPARα, PPARγ, SREBP1c, FAS, and PEPCK (**b**) and for fatty acid oxidation markers (**c**) in liver tissues of E15.5 mice. *p < 0.05 vs. pair-fed mice with saline, **p < 0.01 vs. EtOH-fed mice with saline (n = 8 dams/group). (**d**) Mitochondrial (left) and peroxisomal (right) β-oxidation rates. *p < 0.05 vs. pair-fed mice with saline, **p < 0.01 vs. EtOH-fed mice with saline (n = 8 dams/group). (**e**,**f**) Intracellular ROS production using DCFH-DA (**e**) and mitochondrial membrane potential (ΔΨm) using JC-1 (**f**) in primary hepatocytes isolated from E15.5 mice were measured by using a FACSCaliber flow cytometer. *p < 0.05 vs. pair-fed mice with saline, **p < 0.01 vs. EtOH-fed mice with saline. (**g–i**) The levels of NAD^+^ (**g**) and ATP (**h**) and mitochondrial DNA to nuclear DNA ratio (**i**) in liver tissues of E15.5 mice. *p < 0.05 vs. pair-fed mice with saline, **p < 0.01 vs. EtOH-fed mice with saline (n = 8 dams/group). (**j**) Western blot analysis for mitochondria-encoded COX2 and nuclear-encoded COX-4 protein expression. Data are expressed as the mean ± SEM. Data in (**a–i**) were analyzed by two-way ANOVA with Tukey’s post hoc multiple comparison test. P, Pair-fed; E, Ethanol-fed; FL1, fluorescence channel 1 (JC1 green fluorescence); RFU, relative fluorescence units; NAD, nicotinamide adenine dinucleotide; NAC, N-acetylcysteine.

In ethanol-fed mice, 4-MP administration markedly reduced the increased levels of nonesterified fatty acids (NEFAs) and glycerol, which are produced by ethanol-mediated lipolysis^19^, and C-reactive protein (CRP), an inflammatory marker (Fig. 5a). In line with these observations, the changes in hepatic genes related to lipogenesis (PPAR-γ, SREBP1c, and FAS), fatty acid oxidation (PPAR-α), and phosphoenolpyruvate carboxykinase (PEPCK), the rate-limiting enzyme associated with hepatic gluconeogenesis, in the livers of ethanol-fed mice at E15.5 were reversed by 4-MP (Fig. 5b). Among the genes related to fatty acid oxidation, increased serine palmitoyl-CoA transferase-1 (SPT-1), the rate-limiting enzyme involved in the *de novo* biosynthesis of sphingolipids, and decreased carnitine palmitoyltransferase-1 (CPT-1), which accelerates fatty acid oxidation by transporting fatty acids into the mitochondria, in ethanol-fed mice were markedly rescued by 4-MP treatment (Fig. 5c). The mRNA levels of peroxisomal acyl-coenzyme A oxidase 1 (ACOX1) and long-chain fatty acyl-CoA synthetase (FACS), which are responsible for the initial step in peroxisomal β-oxidation, were significantly downregulated in ethanol-fed mice but were restored by 4-MP. Consistently, treatment with 4-MP markedly rescued the rates of mitochondrial and peroxisomal fatty acid oxidation reduced in ethanol-fed mice (Fig. 5d). Ethanol-induced oxidative stress and lipid peroxidation products are linked to the impairment of mitochondrial oxidative phosphorylation (OXOPHOS), resulting in a reduction in the metabolic rate^20^. As expected, ethanol consumption prior to pregnancy strongly increased ROS production and decreased mitochondrial membrane potential (ΔΨm) (Fig. 5e,f) in primary hepatocytes isolated from maternal E15.5 livers; however, these changes were markedly prevented by 4-MP or antioxidant N-acetyl-cysteine (NAC). 4-MP treatment restored the reduction in nicotinamide adenine dinucleotide (NAD^+^), a classical coenzyme that mediates cellular redox homeostasis, and adenosine triphosphate (ATP) production in the livers of ethanol-fed mice (Fig. 5g,h). The OXPHOS decline in ethanol-fed mice was positively correlated with a significant decrease in mitochondrial DNA, which plays an important role in hepatic respiratory activity and copy number (Fig. 5i), and was correlated with the reduction in cytochrome c oxidase 2 (COX2), a mitochondrial encoded subunit, but not with the reduction in nuclear-encoded COX4; however, the levels were strongly restored by 4-MP (Fig. 5j).

### Ethanol consumption before pregnancy prompts hepatic inflammatory responses

**Figure 6:**
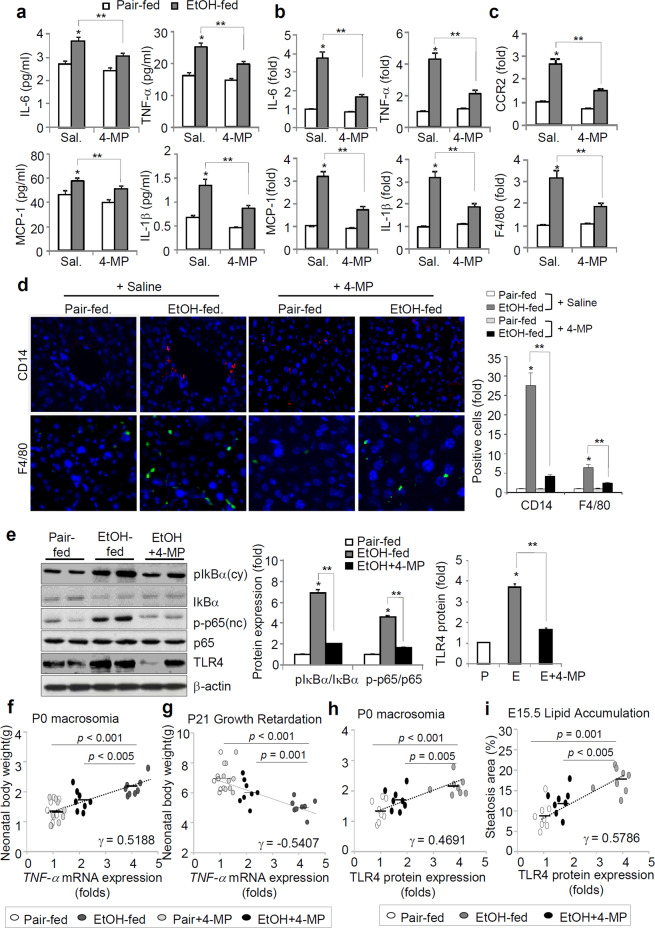
Hepatic inflammatory responses correlate with impaired infant growth and development. The mice were exposed to a liquid ethanol diet for 2 weeks and simultaneously, 4-MP (10 mg/kg, i.p., n = 8/group) was administered three times per week for 2 weeks. (**a**) Serum proinflammatory cytokines for IL-6, TNFα, MCP-1, and IL-1β. *p < 0.05 vs. pair-fed mice with saline, **p < 0.01 (n = 8 dams/group). (**b**) Real-time PCR analyses of hepatic mRNA expression of proinflammatory cytokines in the livers of E15.5 mice. (**c**) Quantitative mRNA analysis of CCR2 and F4/80 in the livers of E15.5 mice. (**b**,**c**) *p < 0.05 vs. pair-fed mice with saline, **p < 0.01 (n = 8 dams/group). (**d**) Representative images of liver sections stained with antibodies against CD14 for monocytes and against F4/80 for macrophages, analyzed by confocal microscopy (scale bars, 10μm) and quantified data. *p < 0.05 vs. pair-fed mice with saline, **p < 0.01 (n = 8 dams/group). (**e**) Representative Western blotting (left) and quantified data (right) for NF-κB activation and TLR4 in livers of E15.5 mice. *p < 0.05 vs. pair-fed mice with saline, **p < 0.01 (n = 8 dams/group). (**f**,**g**) Correlation between *TNF-α* mRNA levels and *p*ostnatal body weight (P0 macrosomia, f) and P21 growth retardation (**g**), respectively. (h and i) Correlation between *TLR4* mRNA levels and P0 macrosomia (**h**) or E15.5 lipid accumulation (**i**). Correlation coefficients (*r*) and *p* values are given. Data are expressed as the mean ± SEM. Data in a-d were analyzed by two-way ANOVA with the post hoc Bonferroni’s multiple comparison test. Data in e were analyzed by one-way ANOVA with Tukey’s post hoc multiple comparison test. Data in f-i were performed by correlation analyses using Pearson’s analysis.

Similarly, the inhibition of ethanol metabolism led to a significant reduction in the mRNA levels of serum and hepatic proinflammatory cytokines (IL-6, TNF-α, MCP-1, and IL-1β) and hepatic inflammatory markers (chemokine (C-C motif) receptor 2 for monocytes and F4/80 for macrophages) that had been elevated by ethanol exposure prior to pregnancy (Fig. 6a–c). Accordingly, immunocytochemistry analyses revealed that 4-MP administration significantly reduced the infiltration of inflammatory CD14-positive monocytes (red) and F4/80-positive macrophages (green) into the maternal livers of ethanol-fed mice (Fig. 6d). However, there were no significant differences between saline- and 4-MP-treated pair-fed mice. The increases in NF-κB activation, which was characterized by an increase in phosphorylated IkBα (cytoplasmic) and the p65 subunit (nuclear), and toll-like receptor 4 (TLR4) expression in ethanol-fed mice were abolished by the inhibition of ethanol metabolism (Fig. 6e). There was a positive correlation between P0 macrosomia and the hepatic TNF-α mRNA levels in ethanol-fed mice at E15.5, whereas offspring growth at P21 was negatively associated with the TNF-α mRNA levels (Fig. 6f,g). Maternal hepatic TLR4 protein expression was positively correlated with P0 macrosomia and hepatic lipid accumulation in the ethanol-fed maternal mouse liver at E15.5 (Fig. 6h,i).

## Discussion

In this study, we provided evidence that ethanol consumption before pregnancy is closely associated with the abnormal development of the pup, including macrosomia and growth retardation, which are correlated with maternal metabolic disorders (Fig. S3). Most previous studies have focused on the detrimental effects of ethanol consumption during pregnancy; however, the effects of ethanol consumption before pregnancy on the progressive development of the fetus and postnatal growth remain obscure. Additionally, few regulatory mechanisms underlying the maternal metabolic disorders induced by ethanol consumption before pregnancy have been identified. Recently, it has been widely accepted that excessive alcohol consumption leads to substantial health concerns, although the moderate drinking offers some health benefits, such as a reduced risk of heart disease and T2DM^21^. In particular, women are more susceptible to the development of alcohol-induced diseases than men^22^, and moderate drinking as well as heavy drinking in women is detrimental to neurogenesis and metabolic homeostasis^23^. However, whether there are negative effects of drinking on women, especially women of childbearing age, remains unclear. Here, compared with pair-fed mice, mice with acute ethanol exposure for 2 weeks before pregnancy led to a marked reduction in the pregnancy rate, embryo number, and average litter size at birth, which reflects a decline in female reproductive function, and attenuated fetal development. Previous studies demonstrated the close relationship between litter size or sex composition and the developmental, survival, and reproductive outcomes of offspring^24^. Other studies also demonstrated that the mean body weights of offspring and litter size were closely associated with the variation of sex composition; however, the overall evidence of such sex-related effects on development remains controversial. In fact, it is well established that alcohol consumption during pregnancy is related to low birth weight, but there is little evidence about the link between alcohol consumption before pregnancy and birth weight. In this study, although the sex of the pups was not confirmed, the reduction in average embryo number was observed at E11.5 of ethanol-fed mice, suggesting the adverse effects of ethanol in the early stage of pregnancy. Therefore, further examination of the links between litter size and sex or size of pups and between litter composition and later life outcomes such as altered development and growth may advance our understanding of adverse effects in ethanol consumption before pregnancy. Maternal metabolic disorders induced by ethanol consumption before pregnancy may affect neonatal macrosomia and growth of the offspring. However, the macrosomia phenomenon was different from a previous report showing that pups from mice who consumed ethanol have a lower birth weight than those of pair-fed control mice^25^. Actually, in humans, fetal birth weight correlates with second- and third-trimester postprandial blood glucose levels but not with fasting or mean glucose levels^26^, suggesting the importance of maternal homeostasis on glucose and insulin tolerance for normal fetal development. In line with this, our data show that mice exposed to ethanol before pregnancy exhibited impaired glucose disposal at the E15.5 phase of pregnancy. Although the data are not shown here, the postprandial blood glucose levels during pregnancy were significantly increased and positively correlated with P0 macrosomia and postnatal growth retardation.

Fetal development and postnatal growth are very complex biological processes that are regulated by both maternal and fetal conditions, including gene regulation and environmental factors^27^. In general, maternal glucose or insulin metabolism plays an important role in the maintenance of metabolic homeostasis and in supplying adequate nutrition for the fetus during pregnancy and fetal development. During normal pregnancy, maternal fasting glucose levels and insulin sensitivity progressively decrease as gestation progresses^28^. Namely, the phenomenon of insulin resistance is observed; insulin resistance helps develop maternal fat depots, which are used as an energy source for the mother and for fetal development. In fact, many studies have demonstrated that alcohol consumption during pregnancy may impair maternal metabolic homeostasis, including alterations in glucose, insulin, or lipid metabolism, thus leading to abnormal fetal development and growth^29^. Additionally, although evidence about the harmful effects of drinking prior to pregnancy on maternal and prenatal health has been continuously accumulating, there is little research on the negative effects of alcohol use prior to pregnancy. Importantly, our data show that ethanol consumption before pregnancy may trigger alterations in maternal metabolic homeostasis during pregnancy. Fasting plasma insulin levels were markedly decreased in ethanol-fed mice at E15.5, whereas glucose disposal tolerance at the acute phase for 2 h after the injection of glucose for GTT analysis was significantly increased. These alterations might be closely associated with a reduction in the ability of pancreatic β-cells to synthesize or secrete insulin, thus causing abnormal maternal glucose homeostasis and hepatic insulin resistance, which consequently contribute to abnormal development of the fetus and offspring. However, the precise reasons or mechanisms related to the increase in the number of offspring with the fused-toe phenotype in ethanol-fed mice are still not known. A previous report suggested that fused toes are an autosomal dominant mouse mutation that interferes with programmed cell death, resulting in fusion of the distal phalanges of the digits of forelimbs^30^. The phenomenon of fused toes was similar to that of syndactyly, which is a digital malformation in which adjacent fingers and/or toes are webbed because they fail to separate during limb development^31,32^. However, even after considerable advances in limb morphogenesis and molecular embryology, it is still not possible to precisely correlate a specific syndactyly/limb anomaly to the underlying genes. Hence, the identification of novel genes for syndactyly could not only elucidate the limb patterning and digit specification mechanisms but also help to single out complex interlocking developmental events. Further studies on the interaction of genetic mechanisms, epigenetic events, pleiotropy and stochastic factors, which generate extreme clinical heterogeneity in affected subjects and families^33^, are needed for the appreciation of fused toes or syndactyly malformation and an earlier prevention or diagnosis of its development.

Several studies have demonstrated that ethanol-induced oxidative stress plays a critical role in organ damage by enhancing oxygen radical or peroxynitrite generation^34,35^. Correlatively, our data provide several lines of evidence showing that pharmacological inhibition of CYP2E1 by 4-MP had therapeutic effects on abnormal fetal development and maternal metabolic disorders in ethanol-fed mice. Interestingly, in normal pregnancy without alcohol intake, CYP2E1 expression in the maternal livers of pair-fed mice was significantly increased compared to expression in the livers of E0 mice, suggesting that pregnant mice experience higher oxidative stress than nonpregnant mice and thus may be more susceptible to the induction of oxidative stress by ethanol. Similar to the detrimental effects of ethanol consumption during pregnancy, our data show that alcohol consumption during the pregestational period may also contribute to maternal metabolic disorders and abnormal fetal development and postnatal growth. Our data also suggest that the oxidative stress generated by ethanol metabolism may be a direct causal factor for impaired maternal metabolic adaptations throughout pregnancy. On the other hand, it is possible that injection or bleeding in dams for GTTs or ITTs may cause considerable stress, which can affect fetal development. To minimize this possibility, GTT and ITT analysis and fasting glucose measurements at E0 and E15.5 were performed in different mouse groups, and after measuring the glucose levels, the mice were sacrificed at E0 and E15.5. Therefore, the mice with postnatal delivery were in different groups from the E0 or E15.5 mouse groups who received GTT or ITT. Our data also show that a blockade of ethanol metabolism strongly attenuated proinflammatory reactions in the livers of ethanol-fed mice, which are closely related to lipid accumulation. Of the many factors that contribute to the pathogenesis of alcoholic liver diseases, gut-derived endotoxin plays a central role in the induction of steatosis, inflammation, and fibrosis in the liver^36^. TLR4 expression, which was upregulated by lipopolysaccharide (LPS), an ethanol-induced endotoxin, was significantly increased at E15.5 in the maternal liver of mice pre-exposed to ethanol before pregnancy and correlated with a significant enhancement of the phosphorylation of NF-κB, p65 and IκB, which are hallmarks of inflammation. Moreover, hepatic TLR4 mRNA expression was positively correlated with P0 macrosomia and lipid accumulation in the maternal liver of ethanol-fed mice at E15.5, suggesting a critical role for the hepatic inflammatory responses increased by maternal alcohol consumption before pregnancy in impairing fetal development and growth. Namely, because the first half of pregnancy is primarily a time of preparation for the demands of rapid fetal growth that occur late in pregnancy, acute or chronic alcohol consumption before pregnancy may affect the first adjustment of maternal nutrient or energy metabolism and may thus trigger oxidative stress-mediated metabolic disorders. All major organs start forming and developing in early stages, which is called the prenatal development period, and thereafter, during the perinatal period, fetal development and maturation are continued, suggesting that the fetal body and organs are developing throughout pregnancy and can be affected by exposure to alcohol at any time. In particular, since the limbs, eyes, and ears are being formed during the fourth week of gestation in humans, the effects of alcohol consumption early in pregnancy can result in defects in these systems and organs. Furthermore, alcohol use in this period is responsible for many of the facial characteristics of fetal alcohol syndrome^37,38^. By the 20^th^ week of gestation, the organs and organ systems are well-formed, and thereafter, the developing fetus and infant are still susceptible to the damaging effects of alcohol. Based on these results, we can suggest that no amount of alcohol is considered safe to drink while pregnant and that there is no safe time point or trimester in pregnancy to drink alcohol, as has been described in a previous report^39^. Additionally, preconception health and health care in Centers for Disease Control and Prevention (CDC) and other health authorities such as US Surgeon Generals’ Advisory statements currently recommend abstaining from alcohol drinking during the preconception period since undertaking to stop drinking before pregnancy reduces the risk of having a baby with a birth defect or that is low birth weight^40,41^.

Unlike normal pregnant dams, mice pre-exposed to ethanol before pregnancy exhibited different regulatory effects on the glucose uptake ability and intracellular glucose disposal responsiveness in early pregnancy. In our study, typical insulin curves did not appear during the GTT performed at E15.5 of pregnant mice. A typical pattern was that the increase in insulin levels during the GTT appeared at 30 min, which was faster than at 60 min after glucose injection, and decreased at 120 min. These discrepancies are thought to be due to the decline of intracellular glucose metabolism activity through a reduction in GCK expression and activity at E15.5 differ to the increase of glucose uptake by increasing Glut2.

The major strengths of this study are that we comprehensively investigated the effects of maternal drinking before pregnancy on impaired fetal development and postnatal macrosomia by using animal models. Moreover, to confirm our results obtained with the animal models, we also analyzed the association of maternal drinking before pregnancy with fetal development and offspring growth using a Korean pregnancy registry database (n = 2,437), although the data are not shown here. Correlatively, our analytical results provided a strong relationship between maternal binge drinking before pregnancy and the development of macrosomia in offspring. Based on these data, we believe that our study is the first to show direct evidence that alcohol intake before pregnancy, not during pregnancy, is closely associated with maternal metabolic disorders and impaired fetal development and macrosomia. Thus, our findings are believed to be useful as basic data for establishing public health policy regarding alcohol drinking. Although our results showed a positive correlation between maternal drinking before pregnancy and postnatal macrosomia via *in vivo* functional studies using mouse models, at present, there are no in-depth mechanisms or approved targets involved in their significant association by using clinical samples such as serum, tissue, or urine. The treatment of macrosomia is rather limited and estimating or predicting a baby’s birth weight is also difficult. A definitive diagnosis of fetal macrosomia and the prognosis of postnatal macrosomia cannot be made until after the baby is born and weighed. Because the prognosis of macrosomia is closely associated with unfavorable in-hospital and serious long-term clinical outcomes, such as metabolic complications including obesity, T2DM, and cardiovascular diseases, as well as growth retardation in the whole life span^42^, a practical and effective solution to these complications is its prevention. Therefore, advances in methods to predict and detect macrosomia earlier are urgently needed. A recent study demonstrated that fetal weight can be estimated based on ultrasound biometry at an early stage of pregnancy, but their positive predictive values for neonatal macrosomia are lower than palpation and symphysial fundal height measurement^43^. In general, maternal diabetes, pregravid weight, parental height, weight gain during pregnancy, age, male sex, prolonged gestation, and multiparity are all positively associated with birthweight, and they are also considered risk factors for macrosomia^44,45^. Furthermore, macrosomic infants have a greater risk of fetal asphyxia, shoulder dystocia, birth trauma and neonatal hypoglycemia^46^, and such infants may have an increased susceptibility to obesity and diabetes later in life^47^. However, little attention has been given to the effects of drinking before pregnancy on the development of neonatal macrosomia. Therefore, it is very important to identify therapeutic target molecules for prepregnancy drinking-induced macrosomia and growth retardation in offspring.

Given the significant association between ethanol metabolism-mediated oxidative stress and maternal metabolic disorders as well as impaired fetal development, drinking before pregnancy may also have deleterious effects on the mother and fetus or offspring. Furthermore, we found that pharmacological inhibition of CYP2E1 or ADH by 4-MP suppressed the maternal metabolic disorders caused by ethanol exposure before pregnancy and subsequently rescued fetal development and growth. Finally, we propose that a public health policy for the reduction or prevention of drinking before pregnancy should be established to ensure maternal and fetal health during all phases of pregnancy, including prepregnancy, pregnancy, lactation, and postweaning.

## Methods

### Animals

Female C57BL/6 mice (6 weeks-old) were obtained from the Jackson Laboratory (Bar Harbor, ME) and subjected to ethanol administration for 2 weeks before pregnancy. The animals were maintained in a temperature-controlled room (22 °C) on a 12:12-h light-dark cycle. Individually caged mice were placed on a Lieber-Decarli Regular Liquid Diet (Dyets) containing 1.0 kcal/ml, of which 18% were derived from protein, 35% from fat, 47% from carbohydrate (control diet, no. 710027) or 11% from carbohydrate and 36% from ethanol (ethanol diet, no. 710260). Ethanol was introduced gradually by increasing the content by 1% (v/v) once in two days until the mice were consuming a diet containing 5% (v/v) ethanol^11,48,49^. Because of the difference in the amount of food consumed in each control diet-fed or ethanol diet-fed mouse (mice consume less ethanol diet than control diet), the control diet-fed mice (n = 8 dams/group) were pair-fed (limited) the same amount of food consumed daily by the ethanol diet-fed mice. The ethanol- or pair-feeding procedures in mice were performed using the National Institute on Alcohol Abuse and Alcoholism (NIAAA) model^11^. Briefly, the feeding tubes with ethanol or control liquid diets were changed in the late afternoon (between 3:00 and 5:00 pm), monitoring the food intake of ethanol-fed mice and calculating the average daily volume per mouse, and then adjusting the volume of control diets accordingly each day. The volume of control diets for the first-day pair feeding is based on the pilot or previous experiments. For C57BL/6 mice, we usually administered 10 ml of control diet per mouse. This diet was then continued for 2 more weeks. In addition, to confirm the effects of ethanol metabolism on alcohol consumption-induced impaired fetal development, 4-methylpyrazole (4-MP; 10 mg/kg maternal body weight), an inhibitor of the ethanol metabolic enzyme CYP2E1, was administered three times per week via intraperitoneal injection for 2 weeks. Following a total of 3 weeks of an ethanol diet, female mice were mated overnight with male C57BL/6 mice (10 weeks old), and pregnancy was immediately confirmed after the first night of mating by a vaginal plug. An additional three days of breeding were performed to obtain enough heads for the experiments. After pregnancy was confirmed, the mice were randomized into four groups (pair-fed, ethanol-fed, pair-fed + 4-MP, and ethanol-fed + 4-MP; n = 36 dams/group) and then sacrificed at each E0, E11.5, and E15.5 stage (n = 8 dams/stage) during pregnancy. The remaining mice were maintained by the delivery stage of P0. The average embryo number, eye developing status, and fetal survival rate were from the mice sacrificed at E11.5 (n = 6 to 9 embryos per dam). The number of mouse used in each group and stage is described in Supplementary Fig. 1a. Delivered dams and postnatal offspring were fed a normal chow diet during the 21-day lactation period, and their body weights were measured at P0, P7, P14, and P21. To measure toe deformities, which are defined as a phenotype with apparent fused toes (*Ft*), toe deformities were measured by counting the number of neonatal (P0) offspring with apparently fused toes. The fused toe (*Ft*) is a digital malformation in which adjacent fingers and/or toes are webbed because they fail to separate during limb development^50,51^. Postnatal body weight in offspring of ethanol-fed and pair-fed mice (n = 8 dams/group) was measured at P0, P7, P14, and P21. Values of postnatal body weight are the mean body weight of all offspring per dam mouse (n = 6 to 9 pups/dam). All animals were cared for in accordance with Korea National Institute of Health guidelines, and all animal experiments were approved by the Korean National Institutes of Health animal care and use committee (Permit Number: KCDC-059-14-2A).

### Measurement of body weight and food intake

Body weight and food intake were measured at least once a week, as well as on the starting day of administration, just before dosing. Food intake was measured once or twice a week.

### The isolation of primary hepatocytes and maintenance

For isolation of primary hepatocytes, mice were anesthetized with pentobarbital sodium (30 mg/kg, IP), and the portal vein was cannulated under aseptic conditions. The liver was perfused first with EGTA solution (5.4 mM KCl, 0.44 mM KH_2_PO_4_, 140 mM NaCl, 0.34 mM Na_2_HPO_4_, 0.5 mM EGTA, and 25 mM Tricine; pH 7.2) and then with DMEM (GIBCO, Gaithersburg, MD) containing 0.075% type I collagenase (Sigma), followed by an additional digestion step (0.009% collagenase at 37 °C with agitation for 15 min) and centrifuged as described previously^52^. The isolated mouse hepatocytes were then cultured in HepatoZYME-SFM media (GIBCO) in mouse-tail collagen-coated plates for 24 h followed by drug treatment.

### Liver histology

The mice were sacrificed after 6 h of fasting. The livers were immediately retrieved and fixed in 10% neutral buffered formalin for 24 h or kept freshly frozen. The fixed liver tissues were embedded in paraffin. We performed hematoxylin and eosin (H&E) staining on paraffin-embedded liver sections (5 μm thickness). Additionally, the liver histology was reviewed for scoring the steatosis area by two experienced liver pathologists according to the guidelines of the Nonalcoholic Steatohepatitis (NASH) Clinical Research Network (CRN) scoring system proposed by Kleiner *et al*.: grading of steatosis, lobular/portal inflammation, hepatocellular ballooning, fibrosis and miscellaneous features^53,54^. We calculated and analyzed the percent area of macro- and microsteatosis in 10 randomly selected histological and anatomical features (images) of each slide per mouse (10 fields of view per mouse, n = 8 dams/group). Ten random fields of steatosis and H&E- and IHC-stained sections at a 10x magnification were assessed in the right lobe of each mouse liver.

### Immunoblots and immunoprecipitation

Cells and tissues were lysed in RIPA buffer at 4 °C, vortexed and centrifuged at 13,000 rpm for 10 min at 4 °C. The supernatant was mixed in Laemmli loading buffer, boiled for 4 min, and then subjected to SDS-PAGE. The separated protein was transferred onto polyvinylidene difluoride membranes, and immunoblotted with primary antibodies and secondary antibodies sequentially. The membranes were incubated at 4 °C overnight with primary antibodies, after which they were washed six times with Tris-buffered saline containing 0.1% Tween-20. The membranes were then incubated with horseradish peroxidase (HRP)-conjugated secondary antibodies for 2 h at room temperature. An anti-pY(941)IRS-1 (44–820 G, 1:1,000) antibody was purchased from Thermo Fisher Scientific. Antibodies against pY(941)IRS-1 (1:1,000), pS(307)IRS-1 (2381, 1:1,000), IRS-1 (2382, 1:1,000), pAKT(Ser473)(9271 S, 1:1,000), Akt (9272, 1:1,000), PGC1α (2178, 1:1,000), SCD1 (2438, 1:1,000), GLUT1(12939, 1:1,000), GCK (3782, 1:1,000), COX2 (12282, 1:1,000), COX4 (4844, 1:1,000), pIκBα (9242, 1:1,000), IκBα (9242, 1:1,000), p-p65 (3039, 1:1,000), p65 (3033, 1:1,000), TLR4 (14358, 1:1,000), and β-actin (4967, 1: 1,000) (all from Cell signaling Technology) were used. Antibodies against PPARα (ab24509, 1: 1,000), FAS (ab133619, 1: 1,000), 4-HNE (ab48506, 1:1,000), and CYP2E1 (ab28146, 1:1,000) were purchased from Abcam. Antibodies against SREBP1 (sc-365513, 1: 1,000) and GLUT2 (sc-518022, 1:1,000) were purchased from Santa Cruz Biotechnology. To increase the specificity, lysates were immunoprecipitated with an antibody against insulin receptor substrate-1 (IRS-1) and then subjected to Western blot analysis.

### Immunohistochemistry and immunocytochemistry

The mice were sacrificed after fasting for 6 h. The livers were immediately retrieved and fixed in 10% neutral buffered formalin for 24 h or kept freshly frozen. The fixed liver tissues were embedded in paraffin. We performed H&E staining on paraffin-embedded liver sections (5 μm thickness). Additionally, immunohistochemistry (IHC) and immunocytochemistry (ICC) analyses were performed. After the liver tissues were fixed, deparaffinized, and washed, the sections were treated with diluted blocking serum containing 5% normal horse serum in TBST for 20 min. Slides were incubated overnight at 4 °C in a humidified chamber with antibodies specific for insulin (4590, 1:250, Cell Signaling Technology.), SREBP1c (sc-365513, 1:250, Santa Cruz), PPAR-α (ab24509, 1:250, Abcam), and CYP2E1 (ab28146, 1:100, Abcam) diluted in 5% blocking serum (goat normal serum, ab7481, Abcam). Sections were incubated with primary antibodies at 4 °C overnight and with a secondary biotinylated antibody against the host species used to generate the primary antibody for 1 h at room temperature. The staining was performed with a Histostain-Plus Kit (Zymed Laboratories, San Francisco, CA) according to the manufacturer’s instructions. Antibody staining was assessed and scored using the “Quick Score method” ^55^. Briefly, the proportion of positive cells was estimated by two of the authors without prior consultation or recourse to clinical, biochemical or previously recorded data. The quick score categories were based on both the intensity and the proportion of brown-stained cells. The category is as follows: scored on a scale of 1 to 6; 0–4% = 1, 5–19% = 2, 20–39% =3, 40–59% = 4, 60–79% = 5, and 80–100% = 6. Anti-CD14 (sc-58951, 1:100, Santa Cruz) and anti-F4/80 (30325, 1:100, Cell Signaling) antibodies were used for ICC. After blocking with 5% BSA in PBS at room temperature for 30 min, slides were incubated overnight at 4 °C with primary antibodies against CD14 and F4/80. The slides were then treated with secondary antibodies (Alexa Fluor-conjugated anti-IgG, Life Technologies) for 30 min at room temperature. After each step, the slides were washed with 0.2% BSA in TBST. Finally, the slides were mounted, and immunofluorescence images were acquired using an Olympus confocal microscope (Olympus, Center Valley, PA, USA).

### Quantitative PCR analysis

Total RNA was isolated from frozen/fresh mouse liver tissues and primary hepatocytes using the Total RNA Purification System (Invitrogen, Carlsbad, CA) according to the manufacturer’s instructions. Reverse transcription was carried out with 1 μg RNA using an iScript cDNA Synthesis Kit (Bio-Rad Laboratories, Hercules, CA). Then, qPCR was performed using SYBR GreenER qPCR SuperMix (Invitrogen) according to the manufacturer’s instructions. mRNA expression levels were normalized to mouse β-actin as the internal standard. The primer pairs for the specific target genes were designed as listed in Supplementary Table S2.

### Lipid peroxidation assays

As a reflection of hepatic levels of lipid peroxidation, the malondialdehyde (MDA) levels were determined by a colorimetric assay (EMD Biosciences, La Jolla, CA). Fifty milligrams of liver tissue was homogenized in 20 mM Tris-HCl, pH 7.4 and 500 μM 3,5-di-tert-butyl-4-hydroxytoluene, and the colorimetric reaction was carried out according to the manufacturer’s instructions.

### ROS assay

Intracellular ROS production was detected using 2’,7’-dichlorodihydrofluorescein diacetate (DCFH-DA; molecular probes, Eugene, OR) at a final concentration of 5 μM according to the manufacturer’s protocol. Following treatment, the cells were washed and incubated with DCFH-DA for 20 min at 37 °C in the dark. The intracellular ROS mediated oxidation of DCF-DA to the fluorescent compound 2’,7’-dichlorofluorescein (DCF). Then, the cells were washed twice with phosphate-buffered saline, and fluorescence was monitored and analyzed by flow cytometry (BD FACSCalibur flow cytometer, FACS101; BIO-RAD Corporation) according to the manufacturer’s instructions.

### Mitochondrial membrane potential

The mitochondrial membrane potential of primary hepatocytes was assessed by using the dual-emission mitochondrial dye JC-1. The cells were incubated with 10 mg/mL JC-1 dye for 30 min at 37 °C and then washed for 5 min in PBS buffer. The percentage of cells with healthy or collapsed mitochondrial membrane potentials was monitored by flow cytometry analysis with an excitation wavelength of 488 nm.

### Determination of the ATP level

Hepatic ATP was estimated by using an ATP Colorimetric/Fluorometric Assay Kit (Abcam, ab83355) according to the manufacturer instructions. Briefly, liver tissue (20–30 mg) was freeze clamped by an aluminum block precooled in liquid nitrogen and was immediately immersed in liquid nitrogen. Liver samples were stored at -80 °C. On the day of the assay, the liver samples were pulverized in liquid nitrogen and homogenized with 6% perchloric acid. Homogenates were centrifuged at 13,000 g for 5 min at 4 °C, and the supernatants were neutralized to pH 7.8 with 2 M KOH, placed on ice for 1 h, and centrifuged at 13,000 g for 5 min at 4 °C. Absorbance was measured at 570 nm after 30 min incubation in a microplate plate reader (Thermo, Multiskan GO). The concentration of ATP was calculated using an ATP standard curve and expressed in nmol/mg protein.

### Measuring mitochondrial DNA

Mitochondrial and nuclear cytosolic fractions were isolated by differential centrifugation, and mtDNA and nuclear DNA were quantified as previously described^56^. Briefly, liver tissues were gently homogenized in 1 ml isolation buffer (225 mM mannitol, 75 mM sucrose, 10 mM 3-(N-morpholino) propanesulfonic acid, 1 mM ethylene glycol tetra acetic acid, and 0.5% bovine serum albumin; pH 7.2). The extracts were then centrifuged at different speeds to obtain cytosolic- and mitochondrial-enriched fractions. For DNA quantification, RNA from the mitochondrial and cytosolic fractions was removed by treatment with RNase A (Thermo Scientific, UK), and the DNA content was determined spectrophotometrically (Nanodrop ND-1000). The DNA concentration in the mitochondrial fraction was normalized to that in the cytosolic fraction to obtain the mtDNA/nuclear DNA ratio.

### Statistical analysis

All results are expressed as the mean ± standard error of the mean (SEM) of the indicated number of independent experiments. Data were examined by the Shapiro-Wilk normality test (<50 samples) or the Kolmogorov-Smirnov test (n ≥ 50) to determine normality. All data exhibited a normal distribution. Data from three or more groups were analyzed by analysis of variance with the post hoc Tukey’s or Bonferroni multiple comparison test. Data for the three groups over time were analyzed by two-way analysis of variance (ANOVA) with post hoc Tukey’s multiple comparison test. For repetitive measurements, we performed a two-way repeated measures ANOVA, followed by post hoc Tukey’s multiple comparison test. Analysis of two samples was performed with two-tailed unpaired Student’s t test, and the statistics for the number of fused toes were calculated using two-tailed Fisher’s exact test. Differences were considered significant at *p* < 0.05. Correlation analyses were performed using Pearson or Spearman’s analysis. The SPSS statistical package (SPSS, Inc., version 18.0, Chicago, IL, USA) was used for all analyses.

## Supplementary information

Supplementary Information.

## Data Availability

The data used to support the findings of this study are available from the corresponding author upon request.
