# Supplementary material for: Alcohol consumption before pregnancy causes detrimental fetal development and maternal metabolic disorders
Source: Sci Rep. 2020 Jun 22;10:10054. doi: 10.1038/s41598-020-66971-1 (PMC7308355; doi:10.1038/s41598-020-66971-1)
Supplement: Supplementary file 1 — Supplementary Information. [file 41598_2020_66971_MOESM1_ESM.docx]

**For Revision into the journal “Scientific Reports”**

**Supplementary Information**

**Alcohol consumption before pregnancy causes detrimental fetal development and maternal metabolic disorders**

Yoo Jeong Lee^2,ǂ^, Ji Yeon Kim^1,2,ǂ^, Dae Yeon Lee^1,2,3,ǂ^, Keon Jae Park^2^, Gyu Hee Kim^2^, Jeong Eun Kim^2^, Gu Seob Roh^4^, Joong Yeon Lim^1^, Seul Koo^1^, Nam Kyoo Lim^1^, Hyun Young Park^1^, and Won-Ho Kim^1,2*^

^1^Division of Cardiovascular Disease, ^2^Division of Endocrine and Metabolic Disease, Center for Biomedical Sciences, Korea National Institutes of Health, Cheongju, Chungbuk, 28159, Republic of Korea; ^3^Department of Biotechnology, Korea University, Seoul, Republic of Korea; ^4^Department of Anatomy and Neurobiology, Gyeongsang National University, Jinju, Gyeongnam, Republic of Korea

***Corresponding author:**

**Won-Ho Kim**; Division of Cardiovascular Disease, Center for Biomedical Sciences, National Institute of Health, #187 Osong Saengmyeong2-ro, Osong-eup, Heungdeok-gu, Cheongju, Chungbuk, 28159, Republic of Korea. Tel.: +82-43-719-8650; fax: +82-43-719-8689. E-mail address: jhkwh@nih.go.kr

**ǂ These authors contributed equally to this work.**

**Supplementary Tables and Figures**

Table S1. Comparison of biochemical parameters in liver tissue or plasma of both pair-fed and ethanol-fed mice for 2 weeks prior to pregnancy.

|  | E0 | |  | E15.5 | |
| --- | --- | --- | --- | --- | --- |
|  | Pair-fed | EtOH-fed |  | Pair-fed | EtOH-fed |
| **Liver tissues** | | | | | |
| Triglycerides (μg/mg) | 64.2 ± 18.1 | 76.4 ± 21.3 |  | 73.6 ± 17.2 | 136.5 ± 41.3**^,&^ |
| Cholesterol (mg/g) | 1.34 ± 0.13 | 1.45 ± 0.21 |  | 1.38 ± 0.32 | 2.28 ± 0.56**^,&^ |
| **Plasma concentration** | | | | | |
| Triglycerides (μg/mg) | 43.2 ± 6.3 | 52.4 ± 8.3 |  | 48.6 ± 9.4 | 77.3 ± 16.1**^,&^ |
| Cholesterol (mg/g) | 128.4 ± 3.9 | 116.2 ± 7.2 |  | 113.5 ± 11.6* | 136.8 ± 8.4**^,&^ |
| Glycerol (mg/dL) | 387.5 ± 49.0 | 448.6 ± 64.7* |  | 403.6 ± 48.2 | 608.5 ± 53.9**^,&^ |
| NEFA (mM) | 0.47 ± 0.07 | 0.63 ± 0.11* |  | 0.41 ± 0.16 | 0.98 ± 0.23**^,&^ |
| Adiponectin (μg/mL) | 43.2 ± 7.2 | 47.5 ± 3.6 |  | 49.4 ± 11.1 | 38.6 ± 4.7**^,&^ |
| IL-6 (pg/mL) | 2.21 ± 0.43 | 2.33 ± 0.74 |  | 2.81 ± 0.32* | 3.72 ± 0.26**^,&^ |
| TNF-α (pg/mL) | 13.4 ± 1.6 | 17.4 ± 2.4* |  | 15.7 ± 1.02* | 27.7 ± 3.04**^,&^ |

Data are expressed as the means ± SD for separated mice groups (n=8 dams/group).

E0, mice on first-day to diagnose the pregnancy; E15.5, mice on day 15.5 of pregnancy; NEFA, nonesterified fatty acids; IL-6, interleukin-6; TNF-α, tumor necrosis factor-α.

*Denotes *p*<0.05 for a significant difference from pair-fed mice at E0; **Denotes *p*<0.05 for a significant difference from pair-fed mice at E15.5 (*p*<0.05); ^&^Denotes *p*<0.01 for a significant difference from ethanol-fed mice at E0.Supplementary Table S2. Primer list used for qPCR.

| Gene name | Accession No | Sequence | Product size(bp) |
| --- | --- | --- | --- |
| SREBP1c | NM_001458314 | Forward 5’-GGAGCCATGGATTGCACATT-3’ | 70 |
|  |  | Reverse 5’-GGCCAGGGAAGTCACTGT-3’ |  |
| FASN | NM_007988 | Forward 5’-AAGCCGTTGGGAGTGAAAGT-3’ | 103 |
|  |  | Reverse 5’-CAATCTGGATGGCAGTGAGG-3’ |  |
| PPARα | NM_011144.6 | Forward 5’-TGCAAACTTGGACTTGAACG-3’ | 105 |
|  |  | Reverse 5’-ATCAGCATCCCGTCTTTGT-3’ |  |
| PGC1α | NM_008904 | Forward 5’-AGCCGTGACCACTGACAACGAG-3’ | 168 |
|  |  | Reverse 5’-GCTGCATGGTTCTGAGTGCTAAG-3’ |  |
| PEPCK | NM_011044 | Forward 5’-ATCATCTTTGGTGGCCGTAG-3’ | 131 |
|  |  | Reverse 5’-ATCTTGCCCTTGTGTTCTGC-3’ |  |
| CPT1 | NM_013495 | Forward 5’-CTCAGTGGGAGCGACTCTTCA-3’ | 105 |
|  |  | Reverse 5’-GGCCTCTGTGTACACGACAA-3’ |  |
| ACOX1 | NM_015729 | Forward 5’-CCACATATGACCCCAAGACC-3’ | 153 |
|  |  | Reverse 5’-AGGCATGTAACCCGTAGCAC-3’ |  |
| IL6 | NM_031168 | Forward 5’-TAGTCCTTCCTACCCCAATTTCC-3’ | 76 |
|  |  | Reverse 5’-TTGGTCCTTAGCCACTCCTTC-3’ |  |
| CCR2 | NM_009915 | Forward 5’-ACACCCTGTTTCGCTGTAG-3’ | 133 |
|  |  | Reverse 5’-GATTCCTGGAAGGTGGTCAA-3’ |  |
| β−actin | NM_007393 | Forward 5’-CACAGCTTCTTTGCAGCTCCT-3’ | 63 |
|  |  | Reverse 5’-GTCATCCATGGCGAACTGG-3’ |  |

SREBP1c: sterol regulatory element-binding protein-1c; FASN: Fatty acid synthase; PPARα: Peroxisome proliferator-activated receptor alpha; PGC1α: Peroxisome proliferator activated receptor gamma coactivator 1 alpha; PEPCK: Phosphoenolpyruvate carboxykinase; CPT1: Carnitine O-palmitoyltransferase 1; ACOX1: Peroxisomal acyl-coenzyme A oxidase 1; IL-6: Interleukin 6; CCR2: C-C chemokine receptor type 2

Supplementary Figure 1


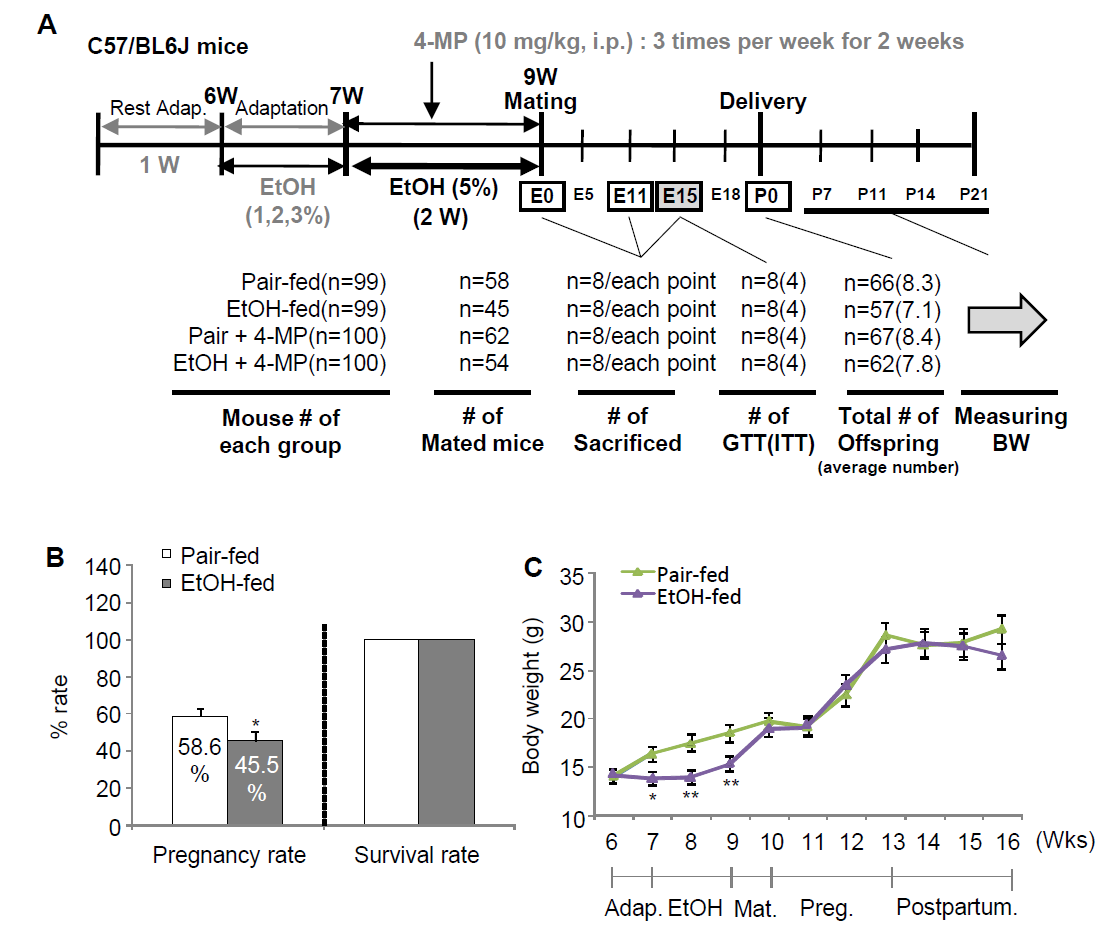


**Fig.S1. Maternal alcohol drinking prior to pregnancy decreases the pregnancy rate and embryo development.** (a) Scheme for pregnancy model pre-exposed to ethanol before pregnancy. Six-week-old female C57BL/6J mice were exposed to a liquid control or ethanol diet for 2 weeks and were impregnated thereafter. Concomitantly, the mice were administered with 4-methylpyrazole (4-MP; 10 mg/kg) three times per week via intraperitoneal injection for 2 weeks. After mating, pregnant mice were randomized into each group. The number (#) of mice used in each group and stage were indicated in the lower panel. (b) Pregnancy and survival rate from mice used in each group (n=8 dams/group). **p*<0.05 from the student *t*-test. (c) Maternal body weight during all phases of the reproductive cycle-prepregnancy, pregnancy, and lactation. **p*<0.05, ***p*<0.01 vs. pair-fed mice (n=8 dams/group), respectively from post hoc analysis for the ANOVA; *p*<0.001 from the ANOVA. Values are means ± SEM. Data in **c** was analyzed by two-way repeated measures ANOVA with Tukey’s post hoc multiple comparison test. Adap., adaptation; Mat., mating; Preg., pregnancy; BW, body weight.

Supplementary Figure 2


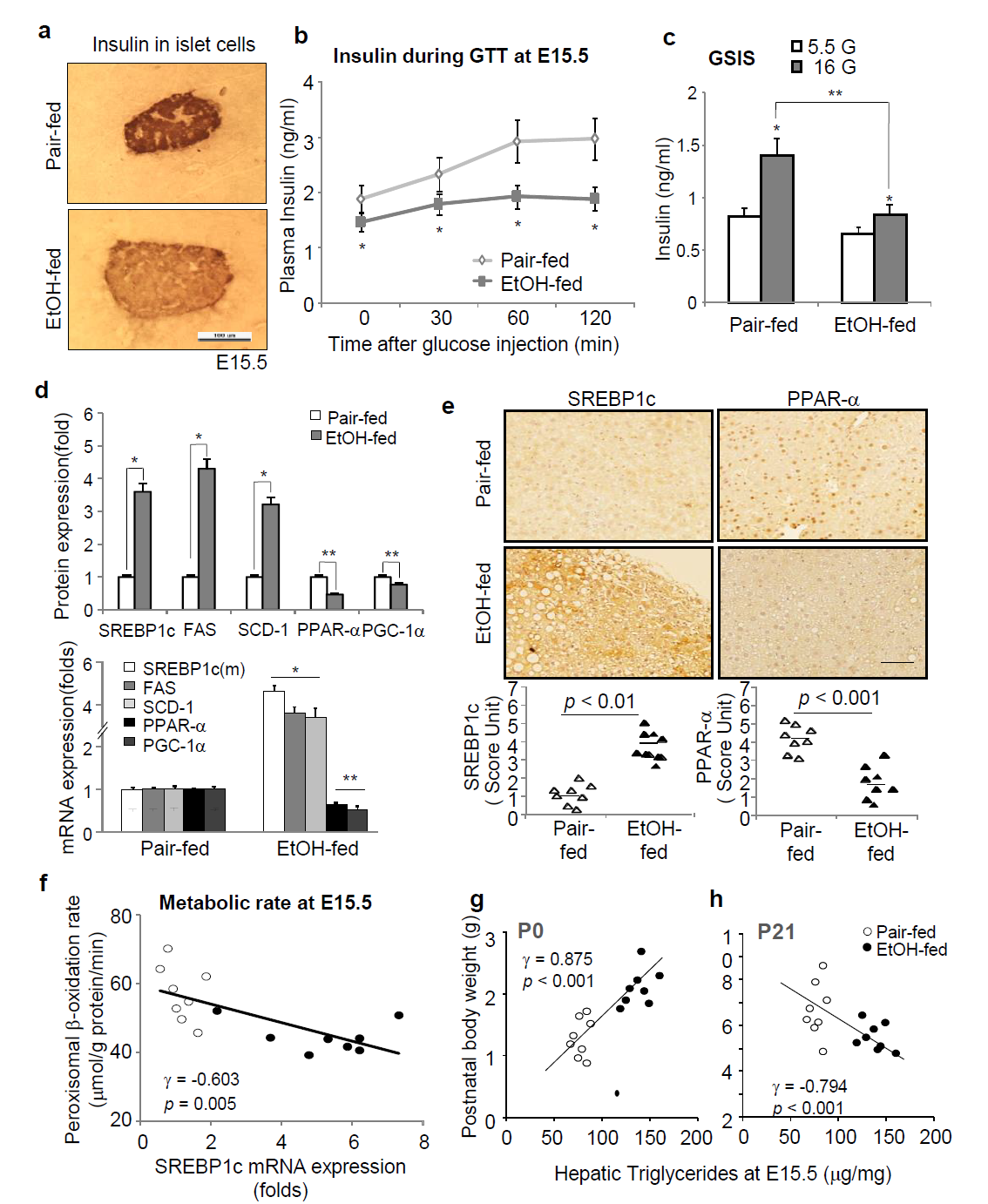


**Fig. S2. Ethanol consumption prior to pregnancy impairs maternal glucose homeostasis during pregnancy.** (a) Immunohistochemistry (IHC) analysis (Scale bar, 100 μm; n=8 dams/group) for insulin in islet cells of pancreatic tissues (E15.5 phase). (b) Plasma insulin levels during GTT at E15.5. **p*<0.01 vs. pair-fed mice from post hoc analysis for the ANOVA; *p*<0.001 from the ANOVA. (c) 16 mM glucose-stimulated insulin secretion (GSIS) in islet cells isolated from pancreas of E15.5 mice. (d) Hepatic expression of mRNAs related to fatty acid oxidation and lipid accumulation by real-time PCR analyses. **p*<0.01, ***p*<0.05 vs. pair-fed mice at E15.5. Data related to Fig 2g. (e) IHC analysis (Scale bar, 50 μm; n=8) of SREBP-1c and PPARα proteins in the liver of E15.5 mice and staining hepatocytes were scored. **p*<0.01 vs. pair-fed mice. (f) Negative correlation between SREBP1c mRNA expression and peroxisomal β-oxidation rate at E15.5. Pearson correlation coefficient (γ) and *p*-value (*p*). (g and h) Correlation between P0 macrosomia (g) or P21 growth retardation (h) and maternal hepatic triglyceride levels at E15.5. Values are means ± SEM. Data in **b** were analyzed by two-way repeated measures ANOVA with Tukey’s post hoc test. Data in **d and e** were analyzed using unpaired Student’s *t* test.

**Supplementary Figure 3**

**
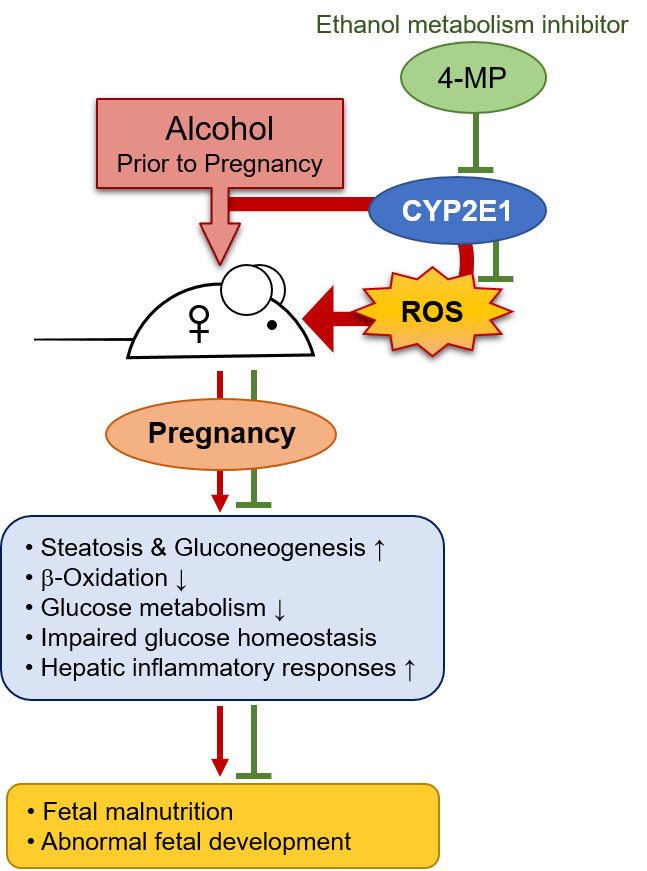
**

**Fig. S3. A proposed model of effects resulting from alcohol consumption before pregnancy.** Alcohol consumption prior to pregnancy, even moderate ethanol feeding, may cause the detrimental fetal development and growth retardation through maternal metabolic disorders. Treatment with 4-MP, an inhibitor of CYP2E1, ameliorate impaired maternal metabolism induced by alcohol consumption before pregnancy.

**Supplementary Methods**

**Biochemical Analysis**

To minimize the impact of maternal stress for fasting glucose, the mice were mildly fasted at 8:00 a.m. for 6h. Blood was collected by cardiac puncture from mice that had fasted for 6 h and then they were sacrificed. Sera and relevant peripheral tissues were stored at −20°C and used subsequently to assess the levels of biochemical parameters. The serum levels of triglyceride and total cholesterol were measured using an enzymatic colorimetric assay based on the GPO-DAOS method and the cholesterol oxidase method, respectively. Hepatic triglycerides and total cholesterol were estimated using commercial diagnostic kits (Sigma). The serum levels of alanine aminotransferase (ALT) and aspartate aminotransferase (AST) were enzymatically measured using a commercial kit from Sigma (Infinity ALT). Serum concentrations of interleukin-6 (IL-6), interferon-gamma (IFN-γ), and tumor necrosis factor-α (TNF-α), and MCP-1 were assessed by ELISA according to the manufacturer’s instructions (Abcam, Eugene).

**Glucose Tolerance Tests (GTT) and Insulin Tolerance Test (ITT)**

For glucose tolerance test and insulin tolerance test, the mice at E0 and E15.5 during the pregnancy were mild fasted at 8:00 a.m_._ for 6 h and then injected intraperitoneally with 1.5 g/kg glucose or 1.5 U/kg regular human insulin, respectively. GTT was performed in each different mouse group at E0 and E15.5 phase and they were sacrificed after measuring the levels of fasting glucose. Basal blood glucose concentrations were determined for each mouse prior to glucose or insulin administration. Blood samples (≤ 5μl) were obtained from the tail vein at time 30, 60, 90, and 120 min after injection, and the blood glucose level was measured using a portable glucose meter (Glucocard II Arkray, Kyoto, Japan)^1,2^. For measuring plasma insulin levels during the GTT, fasting insulin levels were also measured in mildly fasted (6 h) mice and subsequently measured 30, 60, and 120 min post glucose administration using an ultrasensitive mouse insulin ELISA kit (Crystal Chem, Downers Grove, IL) according to the manufacturer’s instruction. On the other hand, to minimize the stress via the injection and bleeding in dams, the analysis for GTT (n=8 dams/group) and ITT (n=4 dams/group) at E15.5 phase carried out on separated mouse groups, respectively, thereafter, the mice were sacrificed at E15.5 phase. Mouse groups applied for analyzing GTT and ITT did not use for other experiments.

**Mitochondrial and peroxisomal β-oxidation**

Mitochondrial and peroxisomal β-oxidation was determined in the postnuclear fraction as acid-soluble products using radiolabeled [I-^14^C] palmitate as a substrate, as described previously^3^. Liver tissues (approximately 1 g) were homogenized in nine volumes of ice-cold sucrose medium (0.25 M sucrose in 10 nM HEPES buffer at pH 7.4, with 1 mM EDTA) and the postnuclear fractions were prepared. Palmitate oxidation rates were measured at 28°C using two media as described previously^4^. The first media allowed the total (mitochondrial and peroxisomal) activities to occur (13.2 mM HEPES at pH 7.3, 16.5 mM MgCl_2_, 82.5 mM KCl, 13.2 mM dithiothreitol, 6.6 mM ADP, 0.2 mM NAD^+^, 100 μM CoA and 0.7 mM EDTA), and the second allowed the peroxisomal activity only (the medium only differing by the presence of 73 mM antimycin and 10 mM rotenone to block the respiratory chain). The palmitate oxidation was measured with 115 μM [I-^14^C] palmitate supplemented with 1.2 mM L-carnitine. The samples were incubated for 30 min at room temperature and the reactions were stopped by addition of 1.5 M KOH; fatty acid-free bovine serum albumin (BSA, 100 mg/mL) was added to the suspension to bind unoxidized substrates, and 4 M HClO_4_ was added to precipitate unoxidized substrates bound to BSA. The total solution was then centrifuged at 1,880 g for 15 min. Aliquots of 200 μL were transferred to a scintillation tube containing 4 mL of liquid scintillation cocktail and assayed for radioactivity in a LS6500 liquid scintillation analyzer (Beckman, USA). Mitochondrial β-oxidation was obtained by subtracting the peroxisomal β-oxidation from the total β-oxidation.

**Determination of liver NAD^+^ levels**

NAD^+^ was determined by a colorimetric method using an NAD^+^/NADH assay kit according to the manufacturer’s instructions (Abcam, Cambridge, MA). Briefly, 20 mg of liver tissues was washed with cold PBS and homogenized (30–50 passages) in 400 μL of NAD^+^/NADH extraction buffer. The supernatant was collected after centrifugation at 14,000 rpm for 5 min at 4°C. The collected supernatant was filtered through a 10 kDa Spin Column (ab93349) to remove the enzymes that rapidly consume NADH before performing the assay. For determination of total NAD^+^ (total NAD^+^t and NADH) and NADH, 50 μL of supernatant was transferred into 96-well plates in triplicates.

**Supplementary References**

1 Kim, J. Y. et al. Chronic ethanol consumption-induced pancreatic {beta}-cell dysfunction and apoptosis through glucokinase nitration and its down-regulation. The Journal of biological chemistry 285, 37251-37262, doi:10.1074/jbc.M110.142315 (2010).

2 Kim, J. Y. et al. Activating Transcription Factor 3 Is a Target Molecule Linking Hepatic Steatosis to Impaired Glucose Homeostasis. Journal of hepatology, doi:10.1016/j.jhep.2017.03.023 (2017).

3. Du, Z. –Y., et al. Biochemical hepatic alterations and body lipid composition in the herbivorous grass carp (Ctenopharyngodon idella) fed high-fat diets. British Journal of Nutrition 95(5), 905-915 (2006).

4. Kim, D. –I., et al. PRMT3 regulates hepatic lipogenesis through direct interaction with LXRα. Diabetes 64(1), 60-71 (2015).

Supplementary Source Data for Western blotting images
